# Towards eco-friendly crop protection: natural deep eutectic solvents and defensive secondary metabolites

**DOI:** 10.1007/s11101-017-9502-8

**Published:** 2017-03-25

**Authors:** Sanae Mouden, Peter G. L. Klinkhamer, Young Hae Choi, Kirsten A. Leiss

**Affiliations:** 1 Research Group Plant Ecology and Phytochemistry, Institute of Biology, Leiden University, P.O. Box 9505, 2300 RA Leiden, The Netherlands; 2 Natural Products Laboratory, Institute of Biology, Leiden University, P.O. Box 9505, 2300 RA Leiden, The Netherlands

**Keywords:** Insecticidal, Metabolomics, NADES, Plant protection compounds, Solubility

## Abstract

With mounting concerns over health and environmental effects of pesticides, the search for environmentally acceptable substitutes has amplified. Plant secondary metabolites appear in the horizon as an attractive solution for green crop protection. This paper reviews the need for changes in the techniques and compounds that, until recently, have been the mainstay for dealing with pest insects. Here we describe and discuss main strategies for selecting plant-derived metabolites as candidates for sustainable agriculture. The second part surveys ten important insecticidal compounds, with special emphasis on those involved in human health. Many of these insecticidal metabolites, however, are crystalline solids with limited solubility which might potentially hamper commercial formulation. As such, we introduce the concept of natural deep eutectic solvents for enhancing solubility and stability of such compounds. The concept, principles and examples of green pest control discussed here offer a new suite of environmental-friendly tools designed to promote and adopt sustainable agriculture.

## Introduction

One of the greatest challenges that agriculture faces in the twenty-first century is the need to feed the world’s rapidly growing population (Hertel 2015). Selection of high-yielding crop varieties have immensely benefitted mankind. However, along with the success of this ‘green revolution’, severe outbreaks of pests and diseases occurred. Agronomic improvements, as a result of domestication, have often been accompanied by limitations such as loss of resistance traits (Wink 1988; Rosenthal and Dirzo 1997). Minimizing crop impairments due to pests has mainly been addressed by the use of synthetic pesticides. Modern agriculture partially owes its success to the discovery and adoption of these chemicals (Cooper and Dobson 2007). Over the past decades, however, concerns have been developed over environmental consequences as well as long-term sustainability. Indiscriminate use of synthetic pesticides has given rise to a number of serious problems, including the widespread development of resistance to pesticides, crop residues, non-target effects, environmental contamination and public concerns about potential health risks (Handford et al. 2015). Furthermore, increased pressure through recent EU legislation (Sustainable Use Directive 2009/128/EC) caused a dramatic shift in pest management strategies, which is not only pushing for tactics that are less reliable on chemicals but, in addition, significantly restrict the application of several important active ingredients (Plant Protection Products Regulation 1107/2009) (European Commission 2009; European Union 2013a, b).

The above facts necessitate the urge for safer, environmentally friendly approaches which, preferably, also exhibit new biochemical modes of action to minimize development of pesticide resistance. Consequently, natural compounds have increasingly become the focus among those interested in discovery of sustainable crop protection agents. This review focuses on plant derived insecticides and discusses their perspectives and challenges as sustainable alternatives in pest management approaches. These naturally occurring bioactive compounds produced by plants, also referred to as secondary metabolites, elicit different insecticidal effects which act as feeding deterrents, growth inhibitors, growth regulators, repellents or oviposition inhibitors against a variety of economically important insect species. Providing a full overview of all plant secondary metabolites with insecticidal potency is beyond the scope here. This review will therefore be deliberately selective, taking a few classes of secondary metabolites of plant origin as examples for natural crop protection, in particular those known to have beneficial health effects on humans.

Though seemingly useful as green insecticides, from a practical perspective secondary metabolites can be a double-edged sword. An inherent problem of many secondary metabolites is their low aqueous solubility, which might hamper commercial formulation. Consequently, organic solvents are often used in large quantities. The need to replace these harmful solvents by safer, non-toxic, inexpensive and easily available ones has significantly increased over the past decades, partially in response to the stringent environmental regulations (Smith et al. 2008). Natural Deep Eutectic Solvents (NADES), a new innovative class of green media, have now come to the fore as such major endeavor.

In this review, we discuss several approaches for selecting plant secondary metabolites as candidates for crop protection. Next, we will briefly focus on the insecticidal properties of a selected sample of plant defense compounds. Finally, NADES are introduced as environmentally benign solvents, which brings a new dimension to the agrochemical industry. In developing this concept, we review the unique solvent properties of NADES and explore their potential as solubilization vehicles for plant derived crop protection agents.

## Secondary metabolites for crop protection

Plants have evolved a variety of defense mechanisms to reduce insect attack, both constitutive and inducible. A key mechanism by which plants defend themselves against attack is through the production of a broad range of secondary metabolites. These represent a large and varied reservoir of chemical structures with many potential uses, including their application as pesticides (Adeyemi 2010; Isman et al. 2011, 2006). Throughout history, numerous plants have been successfully exploited for their pesticidal properties (Thacker 2002). Today, phytochemicals are used to develop commercial insecticides and serve as models for new crop protection agents (Cantrell et al. 2012). Although plant derived biopesticides are generally considered to present lower risks to consumers (Dayan et al. 2009) some plant metabolites such as alkaloids (pyrrolizidines, tropane) as well as certain glucosinolates and saponins are known for their adverse and, possibly even toxic effects (Wiedenfeld and Edgar 2011; Dorne and Fink-Gremmels 2013). On the other hand, there are numerous phytochemicals, particularly phenolic compounds, are associated with human health benefits such as antioxidant, antimutagenic, anti-inflammatory, antimicrobial, antiviral, anti-allergic, immunoprotective and ultraviolet (UV) filtering properties (Dillard and German 2000; Yao et al. 2004). The above benefits in combination with a growing concern about synthetic pesticides make plant secondary metabolites, when carefully selected, highly valuable compounds for crop protection.

## Strategies to identify defensive metabolites

The search for insecticidal biopesticides requires screening of naturally occurring bioactive compounds in plants. Plant secondary metabolites have a wide spectrum of activity against pest insects and are known to affect them at cellular, tissue and organism levels. Thus, it is of utmost importance to study the behavioural patterns in insects to unravel the underlying mechanism responsible for the proclaimed insecticidal activity. Secondary metabolites can have direct implications on insect populations by acute toxicity however, they may also affect population dynamics by impairing important biological traits through physiological and behavioural sublethal effects such as reduced fecundity, malformations and delayed development.

### Metabolomics

Secondary plant metabolites represent a diverse group of low-molecular mass structures which makes comprehensive analysis a difficult analytical challenge (Barah and Bones 2015). Bio-assay guided fractionation is a well-established platform to isolate and characterize active constituents present in plant extracts. However, besides the tedious and time consuming process, another important drawback of this approach is the potential loss of synergistic functions of metabolites during the purification steps. Until recently, studies of phytochemicals were mainly restricted by methods allowing only such reductionist approaches (Hall 2006). In the past decades, a new field of science, known as ‘system biology’, emerged. This holistic approach, collectively placed under the umbrella metabolomics, stands in contrast to the traditional reductionist approach. Metabolomics aims to comprehensively identify and quantify metabolites in a high-throughput, non-biased manner, rather than focusing on a pre-determined small set of metabolites or a specific class of chemical molecules (Kuhlisch and Pohnert 2015).

The significant advances in a variety of analytical platforms have enabled the detection and characterization of such chemically diverse structures. Among them, mass spectrometry in combination with liquid and gas chromatography, as well as nuclear magnetic resonance spectroscopy are most widely used (Aliferis and Chrysayi-Tokousbalides 2011). Each method has its own advantages and limitations in terms of sensitivity, selectivity and reproducibility however, none of them is capable to detect all metabolites within a given biological sample (Verpoorte et al. 2008). Details on analytical technologies used in metabolomics have been reviewed elsewhere (Weckwerth 2003; Allwood et al. 2011; Wolfender et al. 2013).

#### Eco-metabolomics

The last decade has seen an increasing number of applications of metabolomics and has evoked considerable interest in ecological studies including the study of plant-herbivore interactions (Allwood et al. 2008; Macel et al. 2010; Leiss et al. 2011; Maag et al. 2015). Untargeted metabolomics, also known as ‘metabolic fingerprinting’, is well-suited for the discovery of chemical metabolic markers related to plant resistance. Commonly, phenotypic screening is used by analyzing genotypes with contrasting levels of resistance (Jansen et al. 2009; Kuzina et al. 2009; Leiss et al. 2009a, b, 2013; Mirnezhad et al. 2009; Capitani et al. 2012). The next challenge is to make sense of the wealth of data that has been generated during metabolite analysis. Therefore, computational, in conjunction with chemometric or bio-informatic tools, are crucial to process and interpret these results in a biological context (Worley and Powers 2013). The majority of the eco-metabolomic approaches, however, are often correlative in nature. Ultimately, once metabolites are structurally elucidated, their contribution to the observed resistant phenotype needs to be demonstrated in subsequent bioassays. An example of the latter has been provided by Leiss et al. (2009a, 2013), who experimentally addressed the role of several defense metabolites to support the claim of insecticidal activity.

### Insecticidal metabolites

There has been a remarkable interest in the use of biopesticides, specifically plant-based products. This paper presents a critical review of insecticidal metabolites from plant origin, identifying existing challenges as well as opportunities with regards to their use in sustainable crop protection. A literature search was conducted to survey secondary metabolites for their insecticidal properties. After evaluating the available literature over the past two decades, 47 metabolites were selected based on their insecticidal activities. Searches were then carried out for metabolites with proven health-promoting effects. The dual activities of these compounds are highly valuable, both from an ecological and a pharmaceutical perspective. The chosen representatives discussed here include ten metabolites belonging to the phenylpropanoids and flavonoids. Table 1 summarizes their insecticidal activities on various economically important target insects and, where known, their potential mode of action.

**Table 1:** Plant defense secondary metabolites identified for their potential contribution to resistance against various target insect pests

Class	Secondary metabolite	Subclass	Target insect	Main observed effect	Reference
Phenylpronanoids	Caffeic acid	Hydroxycinnamic acid	*Acanthoscelides obtectus* Coleoptera: Bruchidae	Knockdown effect: reduced mobility and increased mortality in adults (in vitro bioassay)	Regnault-Roger et al. (2004)
			*Helicoverpa* zea Lepidoptera: Noctuidae	Prooxidant: oxidative stress in midgut proteins Development rate, survivorship and pupal weight negatively influenced at 4 mM	Summers and Felton (1994)
			*Sitobion avanae* Homoptera: Aphididae	Reduction of phloem sap ingestion at 2.5 mM	Leszczynski et al. (1995)
			*Helicoverpa armigera* Lepidoptera: Noctuidae	Protease inhibitor. Body weight and survival significantly reduced in the range of 50–200 ppm	Joshi et al. (2014)
	Chlorogenic acid	Monolignols	*Dalbulus maidis* Hemiptera: Cicadellidae	Antifeedant activity in adults at 400 ppm (in vitro bioassay)	Dowd and Vega (1996)
			*Plagiodera versicolora* Coleoptera: Chrysomelidae	Antifeedant activity above 50 µg/disk	Jassbi (2003)
			*Agelastica alni* Coleoptera: Chrysomelidae	Feeding deterrent at concentration of 2.48% by weight (dual choice bioassay)	Ikonen et al. (2002)
			*Trichoplusia* *ni* Lepidoptera: Noctuidae	Reduced larval growth and development at 100 ppm (2.8 mM) and 1000 ppm (28 mM)	Beninger et al. (2004)
			*Lymantria dispar* Lepidoptera: Lymantriidae	Reduced larval growth and development at 100 ppm (2.8 mM) and 1000 ppm (28 mM)	Beninger et al. (2004)
			*Spodoptera litura* Lepidoptera: Noctuidae	Developmental retardation and increased larval mortality (>60%) at 0.7–0.8 µg/ml	Mallikarjuna et al. (2004)
			*Frankliniella occidentalis* Thysanoptera: Thripidae	Reduced relative growth rate and survival of first instar larva at 5% (5 mg/ml)	Leiss et al. (2009a)
			*Helicoverpa* zea Lepidoptera: Noctuidae	Prooxidant: chronic exposure at 4 mM inducedoxidative stress in midgut proteins. Development rate and pupal weight are negatively influenced	Summers and Felton (1994)
	Ferulic acid	Monolignols	*Acanthoscelides obtectus* Coleoptera: Bruchidae	Knockdown effect: reduced mobility and increased mortality of adults at day 8 (in vitro bioassay)	Regnault-Roger et al. (2004)
			*Choristoneura fumiferana* Lepidoptera: Tortricidae	Oviposition deterrent 7.8 nmol/cm^2^ (dual choice bioassay)	Grant and Langevin (2002)
			*Sitodiplosis mosellana* Diptera: Cecidomyiidae	Correlation study: positive correlation to resistance. Floret infestation lower in resistant cultivar (Arin cv contains 70.5 mg/100 g DW ferulic acid)	Abdel-Aal et al. (2001)
			*Sesamia nonagrioides* Lepidoptera: Noctuidae	Correlation study: ferulic acid concentration was correlated with the resistance level in the genotypes Structural resistance: cell wall fortification and lignification	Santiago et al. (2006)
			*Rhopalosiphum padi* Homoptera: Aphididae	Constitutive defense of resistant cultivar Regina and induced	Havlickova et al. (1996, 1998)
			*Sitobion avanae* Homoptera: Aphididae	Reduced sieve element salvation at 2.5 mM	Leszczynski et al. (1995)
				Prolonged reproductive period and reduced daily fecundity	Chrzanowski et al. (2012)
			*Sitodiplosis mosellana* Diptera: Cecidomyiidae	Constitutive levels exceeding 0.35 µg/g fresh weight (1.57 µg/g dry weight) increased mortality of newly hatched larvae	Ding et al. (2000)
			*Dalbulus maidis* Hemiptera: Cicadellidae	Antifeedant activity in adults at 400 ppm (in vitro bioassay)	Dowd and Vega (1996)
	p-Coumaric acid	Monolignols	*Etiella zinckenella* Lepidoptera: Pyralidae	Oviposition deterrent at 0.5 mg	Hattori et al. (1992)
			*Rhopalosiphum padi* Homoptera: Aphididae	Constitutive defense; higher concentration in resistant cultivar Regina	Havlickova et al. (1996, 1998)
			*Sesamia nonagrioides* Lepidoptera: Noctuidae	Structural resistance: cell wall fortification and lignification	Santiago et al. (2006)
			*Sitobion avanae* Homoptera: Aphididae	Reduction ingestion of phloem sap	Leszczynski et al. (1995)
			*Dalbulus maidis* Hemiptera: Cicadellidae	Antifeedant activity in adults at 400 ppm (in vitro bioassay)	Dowd and Vega (1996)
	Scopoletin	Coumarin	*Zygogramma exclamationis* Coleoptera: *Chrysomelidae*	Phytoalexin: feeding deterrent. Topical application of 100 µg/ml reduces preference	Olson and Roseland (1991)
			*Sitobion avanae* Homoptera: Aphididae	Reduced ingestion of phloem sap at 2.5 mM	Leszczynski et al. (1995)
			*Plutella xylostella* Lepidoptera: Plutellidae	Antibiosis >2500 µg/g diet growth inhibitor LD_50_ ≈ 8300 µg/ml	Peterson et al. (2003)
			*Spilarctia obliqua* Lepidoptera: Noctuidae	Antifeedant and growth inhibitor. At 250 µg/g deterrence and growth inhibition was similar to that of azadirachtin	Tripathi et al. (2011)
			*Coptotermes formosanus* Isoptera: Rhinotermitidae	Termicidal and antifeedant effects at 5 µmol	Adfa et al. (2010)
			*Anopheles arabiensis* *Diptera*: Culicidae	Repellent activity at 100 µg/ml	Narayanaswamy et al. (2014)
	Sinapic acid	Monolignols	*Choristoneura fumiferana* Lepidoptera: Tortricidae	Oviposition deterrent at 78.6 nmol/cm^2^	Grant and Langevin (2002)
			*Delia radicum* Diptera: Anthomyiidae	Oviposition deterrent; 10 mM in buffered solution reduced number of eggs by 60–70%	Jones et al. (1988)
			*Rhopalosiphum* *padi* Homoptera: Aphididae	Constitutive defense; higher expression correlated with resistance in cultivar Regina (lower reproduction rate). Induction upon feeding on resistant cultivar	Havlickova et al. (1996, 1998)
			*Sitobion avanae* Homoptera: Aphididae	Reduced ingestion of phloem sap at 2.5 mM	Leszczynski et al. (1995)
			*Frankliniella occidentalis* Thysanoptera, Thripidae	Significant increase in mortality of first instar larvae at 25% of natural plant concentrations (0.53 mg/g)	Leiss et al. (2013)
	t-Cinnamic acid	Monolignols	*Mechoris ursulus* Coleoptera: Attelabidae	Contact toxicity: mortality of 86.7% at a rate of 5 mg/paper	Park et al. (2000)
Flavonoid	Luteolin	Flavones	*Acyrthosiphon pisum* Hemiptera: Aphididae	Probing and feeding deterrent at 100 µg cm^−3^	Goławska and Łukasik (2012)
			*Frankliniella occidentalis* Thysanoptera: Thripidae	Significant increase in mortality of first instar larvae at 25% of natural plant concentrations (0.53 mg/g)	Leiss et al. (2013)
	Luteolin-7-glucoside		*Acanthoscelides obtectus* Coleoptera: Bruchidae	Toxin: knockdown effect: reduced mobility and increased mortality	Regnault-Roger et al. (2004)
	Luteolin 7-O- β -D-apiofranosyl-(1→2)-β-D-glucopyranoside		*Liriomyza trifolii* Diptera: Agromyzidae	Ovipositional deterrent at 4.90 µg/cm^2^	Kashiwagi et al. (2005)
	Methyl-luteolin glycoside 2		*Phratora vulgatissima* Coleoptera: Chrysomelidae	Constitutive defense: negative correlation with oviposition in *Salix* F2 hybrids	Torp et al. (2013)
	Rutin	Flavones	*Acanthoscelides obtectus* Coleoptera: Bruchidae	Knockdown effect in bioassay: reduced mobility and increased mortality of adults	Regnault-Roger et al. (2004)
			*Anticarsia gemmatalis* Lepidoptera: Noctuidae	Larval development, amount of food consumed, and pupal weight were negatively influenced at 0.65% (mg/ml)	Salvador et al. (2010)
			*Phyllotreta cruciferae* Coleoptera: Chrysomelidae	Feeding deterrent of 1 month old flea beetle (binary feeding bioassay)	Onyilagha et al. (2012)
			*Spodoptera litura* Lepidoptera: Noctuidae	Developmental retardation and increased larval mortality (>60%) at 0.7–0.8 µg/ml	Mallikarjuna et al. (2004)
			*Lymantria dispar* Lepidoptera: Lymantriidae	Larval growth inhibitor. Larval weight reduced by 45% at 0.05% at day 15	Beninger and Abou-Zaid (1997)
			*Helicoverpa armigera* and *Helicoverpa* zea Lepidoptera: Noctuidae	Feeding deterrent at 10^−3^ M	Simmonds (2003)
			*Helicoverpa armigera* Lepidoptera: Noctuidae	Cessation of feeding inhibits larval growth (1 µg/ml)	Jadhav et al. (2012)
			*Spodoptera litura* Lepidoptera: Noctuidae	Larval development arrested, increased pupal mortality and malformation of adults at 1 µg/ml	Jadhav et al. (2012)
	Kaempferol glycosides	Flavonols	*Frankliniella occidentalis* Thysanoptera: Thripidae	Constitutive defense. Resistance in *Senecio* F2 hybrids correlates with kaempferol-glucoside	Leiss et al. (2009b)
			*Mamestra configurata* Lepidoptera: Noctuidae	Kaempferol-3,7-diglucoside acts as feeding deterrent when applied at concentration > endogenous levels; 0.05–0.45 µmol per leaf disk (choice feeding assay)	Onyilagha et al. (2004)
			*Pieris rassicae* Lepidoptera: Pieridae	Reduced levels of kaempferol 3-O-rhamnoside 7-O-rhamnoside, mediated by overexpression of MYB75, increased susceptibility to feeding. Growth negatively affected when applied exogenously at 100 µM	Onkokesung et al. (2014)
	Quercetin	Flavonols	*Spodoptera litura* Lepidoptera: Noctuidae	Developmental retardation and increased larval mortality (>60%) at 0.7–0.8 µg/ml	Mallikarjuna et al. (2004)
			*Lymantria dispar* Lepidoptera: Lymantriidae	Larval growth inhibitor. Larval weight reduced by 33% at 0.05% at day 15	Beninger and Abou-Zaid (1997)
			*Acyrthosiphon pisum* Hemiptera: Aphididae	Probing and feeding deterrent at 100 µg cm^−3^. At higher concentration mortality and development time are increased whereas fecundity is decreased	Goławska et al. (2014)
			*Spodoptera frugiperda* Lepidoptera: Noctuidae	Feeding deterrent (57%) at 100 µg/cm^2^	Diaz Napal and Palacios (2015)
				78% Larval mortality at 0.1 mg/g	Gallo et al. (2006)
			*Helicoverpa armigera* Lepidoptera: Noctuidae	Negative effect on growth reducing larval weight by ~50% at 16 mg/g development reducing pupation by 50% at 8 mg/g) and survival (>80% mortality at 32 mg/g)	Li et al. (2016)
				Deleterious effects growth, pupation and adult emergence, and survivalat 3 and 10 mg/g	Liu et al. (2015)
			*Bactrocera Cucurbitae* Diptera: Tephritidae	Growth regulator and or deterrent: reduction in food assimilation as well as larval and pupal weight at 125 ppm	Sharma and Sohal (2013)
			*Aphis fabae* Hemiptera: Aphididae	Inhibitory activity on progeny deposition 0.1 M	Lattanzio et al. (2000)

#### Phenylpropanoids

Phenylpropanoids, are among the most common and widespread plant secondary metabolites involved in plant defense (Dixon et al. 2002). The biosynthesis of phenylpropanoids originates from phenylalanine. Followed by sequential hydroxylation and methylation reactions of *trans*-cinnamic acid, several substituted derivatives such as *p*-coumaric acid, caffeic acid, ferulic acid, chlorogenic acid and sinapic acid have frequently been implicated in plant defense against insect herbivores, including, Hemiptera, Lepidoptera, Orthoptera, Coleoptera, Thysanoptera and Diptera.

Phenylpropanoids often function as feeding deterrents and digestibility reducers (Table 1). Cell wall modifications, mainly established by hydroxycinnamic acid derivatives, may directly pose physical barriers to various insect species incorporating and cross-linking with carbohydrates (Abdel-Aal et al. 2001; Santiago et al. 2006; Leiss et al. 2013). Phenols act as pro-oxidants (Summers and Felton 1994) whereby their oxidative products covalently bind to amino acids and proteins decreasing the digestibility of dietary proteins (Felton et al. 1992). In addition, the insecticidal activity of phenols also arises from inhibition of vital insect gut proteases as has been shown for caffeic, ferulic, sinapic, chlorogenic and *p*-coumaric acid (Johnson 2005; Joshi et al. 2014).

#### Flavonoids

Flavonoids represent one of the most studied classes of phenylpropanoid-derived metabolites and are found ubiquitously in plants (Harborne 2001; Simmonds 2001). Structurally, flavonoids consist of several classes such as flavones (e.g., luteolin, rutin), flavonols (e.g., kaempferol, quercetin), flavanones (e.g., naringenin) and others.

Flavonoids have many complex roles in plant–insect interactions (Simmonds 2001). A number of flavonoids and some glycosides have been investigated as feeding deterrents, digestibility reducers or as metabolic toxins against many insect pests (Treutter 2006; Mierziak et al. 2014). Negative effects of flavonoids on herbivore survival as well as performance including growth and fecundity have been demonstrated by artificial diet experiments or *in planta*. Rutin and quercetin represent model phenolics in the study of plant defense compounds due to their abundant occurrence and well documented toxicity to numerous insect herbivores. However, despite the importance of flavonoids in plant–insect interactions, detailed understanding of how they modulate resistance at the biochemical and molecular levels remains largely unknown (Simmonds 2003).

As with many chemicals, the dosage often determines the degree of effect it produces. Depending on the insect species, rutin and quercetin, at varying doses, elicited variable behavioral responses and provoked both negative as well as stimulating effects on herbivore feeding (Simmonds 2003; Jadhav et al. 2012; Golawska and Lukasik 2012; Diaz Napal and Palacios 2015). Another level of complexity is posed by the fact that defense responses to a specific compound can often be modulated by the presence of other compounds. For example, methanol extracts of *Lonicera maackii*, dominant in the flavonoid luteolin, deterred feeding of the generalist herbivore *Spodoptera exigua*. However, when offered as individual compound in diet plugs, no anti-herbivory activity was observed. Instead, luteolin was marginally stimulating feeding (Cipollini et al. 2008). Onyilagha et al. (2012) provided strong evidence of defensive synergies among different flavonoids. While individual metabolites minimally deterred flea beetle feeding, combined flavonoid fractions were more effective in feeding deterrence. This highlights the importance of complex matrices of plant extracts in which active substituents might act additively or synergistically.

### Secondary metabolites: when poor solubility becomes an issue

Plant secondary metabolites represent a new generation of green insecticides with potential opportunity for commercial utility in agriculture (Adeyemi 2010; Dayan et al. 2009). However, the majority of secondary metabolites are poorly water soluble, thus limiting their application as crop protection agents. The results of our literature search indicate that 78% of the selected insecticidal metabolites are poorly water soluble. For products such as pharmaceuticals, efficacy often depends on the effective solubility of the active ingredient to ensure proper dispersion. Solubility, therefore, presents a key prerequisite for ensuring successful formulation. Numerous candidates fail to reach commercialization due to solubility problems (Lipinski 2002). The literature on solubility issues regarding agrochemicals is relatively scarce, however, we can safely assume that the same dilemma exists there. To illustrate this statement, we summarized the aqueous solubilities of the ten representative metabolites in Table 2. Discrepancies of water solubility measurements reported in the literature may be attributed to one or more of the following: compound purity, particle size, analytical method employed or time allowed for equilibrium conditions to be reached. As such, the reported water solubilities were retrieved from a database using ALOPGS 2.1 as a modeling software program predicting aqueous solubility.

**Table 2:** Solubility data of plant secondary metabolites in water

Designation	Secondary metabolite	Solubility (mg/ml)
Very slightly soluble	Ferulic acid	0.906
	Kaempferol	0.178
	Luteolin	0.138
	Sinapic acid	0.631
	Quercetin	0.261
Slightly soluble	Caffeic acid	1.61
	Chlorogenic acid	3.44
	*t*-Cinnamic acid	1.15
	*p*-Coumaric acid	1.02
	Rutin	3.54
	Scopoletin	2.35

Data retrieved from ALOPGS 2.1. According to the United Stated Pharmacopeia (USP30), the water solubility of a ‘slightly soluble’ compound ranges from 10 mg/ml down to 1 mg/ml whereas ‘very slightly soluble’ compounds are defined as 1 mg/ml to 100 µg/ml

Descriptive terms are often used to designate solubility and usually refer to ranges of solubility rather than providing detailed information on true solubilities. According to the United Stated Pharmacopeia (USP30), the water solubility of a ‘slightly soluble’ compound ranges from 10 mg/ml down to 1 mg/ml whereas ‘very slightly soluble’ compounds are defined as 1 mg/ml to 100 µg/ml. In addition, interpretation of the term ‘poorly-soluble compound’ can vary, depending on an individual’s definition. Therefore, the term low solubility in this review is defined as the aqueous solubility of a compound that falls into the range of ‘slightly soluble’ and below (i.e. <10 mg/ml). There are various methodologies available to enhance the aqueous solubility of chemical compounds (Savjani et al. 2012). In this paper, we discuss the potential of a recently developed green solvent, known as NADES, to improve the solubility of poorly soluble defensive metabolites.

### Deep eutectic solvents

**Fig. 1:**
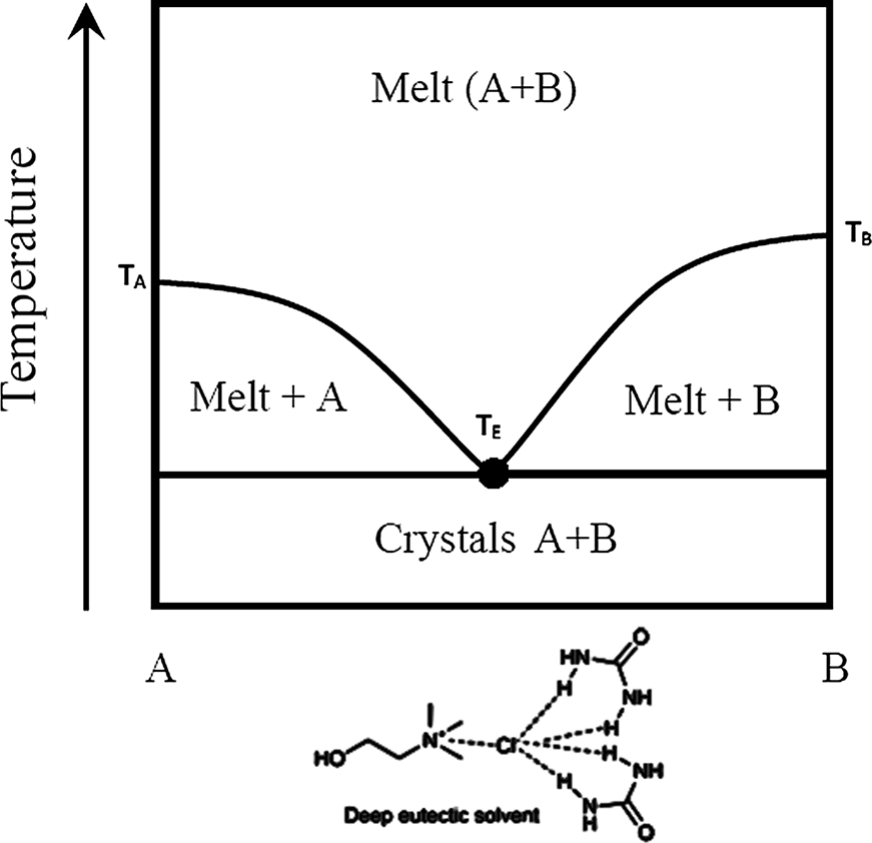
Schematic phase diagram of binary chemical mixture. Urea (A), at the far *left* of the diagram has melting temperature (T_A_) of 133 °C. Choline chloride (B), at the far *right* of the diagram has a melting temperature (T_B_) of 302 °C. The liquidus lines slope downwards the melting points of the pure components and meet at a point known as the eutectic point (indicated by the *black dot*). Deep eutectic liquids (T_E_) have melting points far *below* any of the starting materials

As with many conventional pesticides, a low aqueous solubility often requires large amounts of organic solvents (e.g., alcohols, chlorinated hydrocarbons, arenes, and nitriles) to be used in agrochemical formulations (Anjali et al. 2010). In most cases, active ingredients of biopesticides are formulated in a similar way (Gasic and Tanovic 2013). Considerable attention has been directed towards the reduction or elimination of organic solvents for safer handling (Knowles 2008; EEA 2013). This increasing environmental consciousness has led to the development of greener formulations as alternatives to hazardous organic solvents. The motivation to develop solvents that are less harmful to the environment became more apparent with the development of ionic liquids. Ionic liquids (ILs) are liquids that are entirely composed of ions with melting points lower than 100 °C (Ruß and König 2012). For a long time ILs were hailed as green solvents for the future. ILs, however, encounter several drawbacks such as toxicity and low-biodegradability questioning their ‘greenness’ (Paiva et al. 2014). In the search for green alternatives, deep eutectic solvents (DES), emerged as a promising substitute for both ILs as well as organic solvents (Alonso et al. 2016). The term DES was first introduced by Abbott more than a decade ago (Abbott et al. 2003). His pioneer work led to the discovery of liquids with unique physicochemical properties that were obtained by mixing two solids. A classical example is the mixture of urea (melting point 133 °C) with choline chloride (melting point 302 °C). Figure 1 illustrates the phase diagram of the urea-choline chloride system. At a molar ratio of 2:1 (urea-choline chloride) a eutectic mixture is formed at 12 °C.

As the research interests in deep eutectic solvents grew in the past decade, many innovative applications of DES emerged including, among others, in analytical chemistry (Karimi et al. 2015; Zheng et al. 2014) biotechnology (de María and Maugeri 2011), extraction and separation processes (Bi et al. 2013; Dai et al. 2013), nanotechnology (Abo-Hamad et al. 2015), drug delivery (Morrison et al. 2009; Aroso et al. 2016), polymerization (del Monte et al. 2014) and electrochemistry (Nkuku and LeSuer 2007).

The field of DES is growing at a fast rate, with an increased interest in natural and bio-renewable compounds (Espino et al. 2016). Following the pioneering work of Abbot, the concept of DES was extended to numerous combinations of natural origin (Abbott et al. 2004; Imperato et al. 2005; Choi et al. 2011; Ruß and König 2012). More recently, a wide range of bio-based compounds for constructing a new class of innovative green solvents, known as natural deep eutectic solvents (NADESs) were discovered (Choi et al. 2011). This revolutionary class of non-toxic media provides a novel biotechnological solution to deal with scarcely water soluble metabolites.

## Natural deep eutectic solvents

NADES are commonly based on naturally occurring plant compounds, in particular primary metabolites. It has been hypothesized that, in analogy to synthetic ionic liquids, living organisms contain a third liquid medium as an alternative to water and lipids. This hypothesis arose from the observation that many solid primary metabolites became liquid when mixed in a certain ratio, suggesting that DES had long been invented by nature itself. The occurrence of NADES, intracellularly, helps to explain certain biochemical processes such as the biosynthesis, storage and transport of compounds which otherwise would be difficult to solubilize (Choi et al. 2011). More than 100 stable combinations of NADES were designed, based on particular molar ratios of two compounds such as amino acids, organic acids, sugars or choline derivatives (Dai et al. 2013). Water is often incorporated as a third component and is strongly retained in the solvent (Choi et al. 2011). Due to the generation of intramolecular hydrogen bonds the resulting NADES displays a high melting point depression causing the solids to liquefy. This eutectic mixture, which is characterized with a melting point temperature that is far below its individual precursors, remains fluid at room temperature.

### NADES as designer solvents

Modifying the nature and molar ratio of the compounds allows to customize these properties in order to meet specific requirements hence, the accolade ‘designer solvents’. The plethora of possible combinations can therefore, be seen as a huge opportunity to find a suitable solvent for any application (Francisco et al. 2013). Choline chloride, an inexpensive, non-toxic, and biodegradable quaternary ammonium salt, is by far one of the most dominant constituents used in the field of deep eutectic solvents. Previously known as vitamin B_4_, it has some important key functions in the human body. Choline chloride serves as building block for membrane phospholipids and as precursor of the neurotransmitter acetylcholine (Ueland 2011).

Among the available preparation methods, the heating and stirring method is the most common one for preparing eutectic solvents (Espino et al. 2016). This easy method simply requires mechanical stirring of solid starting materials while heating at moderate temperature until a homogenous liquid is formed. Besides the ease of preparation, NADES offer several other advantageous qualities as solvents such as a wide liquid range, water compatibility, low toxicity, non-flammability, biocompatibility and low vapor pressure (Paiva et al. 2014).

#### Physicochemical properties of NADES

Being designer solvents, the physicochemical properties of NADES, e.g. melting point, density, viscosity, acidity, and hydrophobicity, are highly tunable (Dai et al. 2015). Solvent polarity is an important parameter in chemistry that characterizes how a solvent interacts with solutes. Typically, solvents can be classified into three main categories: non-polar solvents (hydrocarbons), polar protic solvents (e.g. water, alcohols) and polar aprotic solvents (e.g. DMSO, acetone). The solvation properties of NADES cover a wide range of polarity. Mixtures of organic acids were most polar, followed by amino acids based NADES whereas polyalcohol based NADES are least polar, displaying a polarity similar to that of methanol (Dai et al. 2013). Nonetheless, the majority of NADES are generally hydrophilic due to their hydrogen bonding ability. Consequently, hydrophobic eutectic mixtures based on menthol and fatty acids have now come to the fore (Ribeiro et al. 2015; van Osch et al. 2015). Another important physical property is viscosity. NADES tend to be fairly viscous when compared to traditional organic solvents, which forms a disadvantage for practical applications. The strong hydrogen interactions, which are the key to NADES formation, promote these high viscosities. Both properties, polarity and viscosity, may be modulated by the addition of water (Dai et al. 2013). The viscosity of NADES is significantly decreased upon dilution with water, while still maintaining its supermolecular structure (Dai et al. 2015).

### Green defense against pests

Driven by legislation and evolving societal attitudes concerning environmental issues, the search for safe and green products has been increasing continuously. As a contribution to such efforts, we present an alternative green approach which involves the use of insecticidal crop protection agents and solvents from plant-origin.

### Green formulations: improving solubility with NADES

Chemical formulations, especially during earlier phases of research and development, mostly start with the evaluation of their general suitability prior to launching into full development (Sasson et al. 2007). One of the most frequently asked questions that scientist face in technical fields relating to chemical formulation of compounds concerns the solubility of a specific active ingredient (Battachar et al. 2006). While at first glance the answer seems to be just a simple number, it is one of the most critical pre-formulation parameters that has a significant impact on the performance of a molecule. In this paper, we demonstrated the potential of NADES as a promising sustainable solvent for improving the solubility of several resistant related secondary metabolites.

NADES has several important advantages, particularly the high solubilizing capacity of both polar and non-polar compounds. The solubility of the poorly soluble insecticidal metabolites rutin, quercetin and *trans*-cinnamic acid was significantly increased as compared to the aqueous solubility (Dai et al. 2013, 2015). The strong hydrogen bonding between NADES and the solutes did not only cause this huge increase in solubility but also contributed to the stability of secondary metabolites under various conditions such as high temperature, light and storage time (Dai et al. 2013).

As an extension of these studies, we have investigated the solubility of six insecticidal metabolites in a variety of NADES. The solvent selection framework consisted of the following steps:

1. Preliminary solvent screening: a pre-selected set of 20 NADES was used as a starting point to identify and rank potential NADES candidates (Table 3). Selection constraints were imposed on important properties such as viscosity and stability.

**Table 3:** Different combinations of natural deep eutectic solvents

	NADES components	Molar ratio
1	1,2-propanediol: choline chloride: water	1:1:1
2	1,2-propanediol: choline chloride: water	1:1:3
3	β-Alanine: Citric Acid: water	1:1:3
4	Betaine: citric acid: water	1:1:5
5	Fructose: chloline chloride: water	1:1:3
6	Glucose: choline chloride: water	2:5:5
7	Glucose: citric acid: water	1:1:5
8	Glycerol: choline chloride: water	2:1:1
9	Lactic acid: choline chloride	1:1
10	Lactic acid: 1,2-propanediol	1:1
11	Lactic acid: 1,2-propanediol	2:1
12	Lactic acid: glucose: water	5:1:3
13	Lactic acid: β-Alanine: water	1:1:3
14	Malic acid: sorbitol: water	1:1:3
15	Malic acid: l-serine: water	1:1:3
16	Malic acid: choline chloride: water	1:1:2
17	Proline: malonic acid: water	1:1:6
18	Xylitol: choline chloride: water	1:1:2
19	Xylitol: choline chloride: water	1:2:3
20	Xylitol: citric acid: water	1:1:3
2. Secondary screening: solubility tests were performed to determine the best solvent for each of the metabolites. Saturated solutions, generated by adding an excess amount of each compound to different NADES, were kept under constant stirring (1100 rpm) for 24 h at 50 °C. The 20 candidates were ranked in decreasing order of solvent power.
3. Solubility verification: For the verification of solubilities, samples were centrifuged for 5 min at 2000 rpm and subsequently diluted suitably with methanol for spectrophotometric analysis (UV-1800 UV–VIS spectrophotometer, Shimadzu Europe GmbH, Duisburg, Germany). Five different concentrations, in the range of 3–35 μg/ml, were prepared in triplicate to construct a standard curve. Corresponding solubilities, analysis wavelengths, linear range and correlation coefficient (*r* ^2^) are presented in Table 4.

**Table 4:** Solubility of insecticidal metabolites (mg/g) in different natural deep eutectic solvents

Secondary metabolite^a^	NADES composition	Water percentage	λ max (nm)	Linearity range (μg/ml)	Correlation coefficient	Solubility (mg/g)
Sinapic acid	lactic acid: 1,2-propanediol (2:1)	–	325	3–12.5	0.9981	21.59 ± 0.24
Chlorogenic acid	lactic acid: 1,2-propanediol (2:1)	–	325	3–20	0.9994	100.92 ± 0.57
Luteolin	1,2-propanediol: choline chloride: water (1:1:1)	7.71%	348	3–15	0.9941	13.08 ± 0.86
Rutin	1,2-propanediol: choline chloride: water (1:1:3)	20.04%	359	3–35	0.9961	18.46 ± 0.67
Quercetin	1,2-propanediol: choline chloride: water (1:1:1)	7.71%	375	3–20	0.9975	44.34 ± 0.76
Ferulic acid	lactic acid: 1,2-propanediol (2:1)	–	322	3–12.5	0.9901	31.92 ± 0.70

Data represented as mean ± SD, n = 3

^a^Luteolin was purchased from Chengdu pharmaceutical co ltd (Chengdu, China), whereas all other metabolites were obtained from Sigma (MO, St. Louis, USA)

Among the pre-screen solvents, lactic acid: 1,2-propanediol in a molar ratio of 2:1 (abbreviated by LAP 2:1) and 1,2-propanediol: choline chloride: water in molar ratios of 1:1:1 and 1:1:3 (abbreviated PCH 1:1:1 or 1:1:3) have demonstrated considerable improvement in solubility. Results of solubility studies indicated that, enhancements with NADES, as compared to aqueous solubility, were more than 29, 34, 35, 95 and 195 fold in cases of chlorogenic acid, sinapic acid, ferulic acid, luteolin and quercetin, respectively.

A major advantage of the high solubilizing power is that it allows a high degree of flexibility in tailoring dosage treatments. However, an important drawback of NADES that might constrain the applicability, is its’ high viscosity. While modifiers such as water can be used to reduce the high viscosity, it also significantly affects the solubility (Dai et al. 2013). The solubility of rutin, for example, was increased by fivefold in PCH (1:1:3) compared to water (Tables 2, 4). For nonpolar compounds, the highest solubility is achieved in pure NADES, whereas solutes with a medium polarity such as rutin display a higher solubility when diluted with 5–10% of water. Increasing the water content to 25 or 50% (v/v) drastically reduced the solubility, which presumably is attributed to the loss of the supermolecular structure of NADES (Dai et al. 2013) and provides an explanation of the less pronounced enhancement of rutin in PCH (1:1:3). Interestingly, the insecticidal metabolites trans-cinnamic acid, caffeic acid and p-coumaric acid can also be included as hydrogen bond donors for the formation of NADES (Maugeri and de María 2012).

### Concluding remarks: the way forward for sustainable agriculture

The pest control industry is constantly searching for innovative approaches that advance the way we manage pest insects. This emerging need has created a significant market opportunity for alternative and bioactive products such as plant derived metabolites. The interest in phytochemicals extends beyond their natural function as defensive weapons against insect attack as many appear to provide numerous desirable health benefits. Central to the control of pest insects is the question of how these green substituents can be formulated and promulgated. The current review, therefore, presents a simplified guide from plants to practice. This approach comprises the following three essential elements: (1) a robust, reliable and quantitative eco-metabolic approach to screen for bioactive metabolites, (2) a rigorous validation process to study and verify the insecticidal activity, and (3) a strategy for improving the solubility of sparingly soluble compounds.

With increasing pressures on product performance, formulation is a key technology for agrochemical companies to differentiate their products and add significant value. As such, NADES are introduced as environmentally benign solvents presenting a promising solution to enhance the solubilizing properties of poorly-soluble insecticidal metabolites.

Drug delivery systems based on eutectic mixtures have been described to increase drug bioavailability (Aroso et al. 2015, 2016). As for the implementation of these green alternatives, efforts should be made in evaluating NADES as a solvent carrier system for the delivery of these insecticidal compounds. The application of insecticidal plant secondary metabolites as a pre-sowing treatment for seeds (e.g., coating) and cuttings (e.g., dipping) presents a promising approach to protect plants from their most vulnerable stage onwards. Rather than shying away from unknown challenges presented by new technologies, we should take the opportunity to use and develop them to improve pest control strategies. Those concerned with developing sustainable crop protection agents are therefore, highly encouraged to assess the applicability of these plant-derived alternatives.
